# Study on the Fatigue Bending Strength of Cylindrical Components Manufactured by External WAAM

**DOI:** 10.3390/ma18204791

**Published:** 2025-10-20

**Authors:** Van-Minh Nguyen, Pham Son Minh, Dang Thu Thi Phan, Huynh Do Song Toan

**Affiliations:** Faculty of Mechanical Engineering, HCMC University of Technology and Education, Ho Chi Minh City 71307, Vietnam; minhps@hcmute.edu.vn (P.S.M.); thuptd@hcmute.edu.vn (D.T.T.P.); toanhds@hcmute.edu.vn (H.D.S.T.)

**Keywords:** wire arc additive manufacturing (WAAM), fatigue bending strength, Taguchi method, process parameters, path strategy, MIG welding, CT3 steel, fatigue life, additive manufacturing, optimization

## Abstract

This study investigates the fatigue bending strength, measured as the Mean of Fatigue Cycles (N), of cylindrical components produced by external wire arc additive manufacturing (WAAM) through a Taguchi L25 orthogonal array and linear regression analysis. Five welding parameters—welding current (Ampe), offset distance (mm), step length (mm), welding speed (mm/min), and specimen gauge diameter (mm)—were evaluated to maximize N using signal-to-noise (S/N) ratios. Nominal bending stresses (σ) ranged from 45 to 54 MPa, ANOVA on raw replicate data (25 runs, 3 replicates) confirmed specimen gauge diameter = 17 mm and weld current = 125 A as dominant, with F = 171.62 (*p* < 0.001, eta^2^ = 0.62 [95% confidence intervals (CI) 0.55–0.68]) for specimen gauge diameter and F = 6.13 (*p* < 0.001, eta^2^ = 0.13 [95% CI 0.08–0.18]) for weld current, accounting for ~75% of the variance. Optimal settings (offset distance = 3.0 mm, step length = 1000 mm, welding speed = 550 mm/min, specimen gauge diameter = 17 mm) achieved S/N = 111.35 dB, predicting N ≈ 350,000–380,000 cycles, a 22–33% improvement. Interactions between specimen gauge diameter and speed, and between weld current and offset distance, suggested enhanced strength at speed = 400–450 mm/min for specimen gauge diameter = 17 mm. Basquin’s law (b ≈ 0.72, R^2^ = 0.992) confirmed weld current as key. The linear regression model (adjusted R^2^ = 0.9506) had coefficients for specimen gauge diameter (+70,120 cycles/mm, *p* < 0.001) and weld current (+1088 cycles/Ampe, *p* = 0.02), but lower test R^2^ = 0.7212 via cross-validation (60/20/20 split) indicates overfitting due to small dataset size (25 runs), suggesting larger datasets or nonlinear models (e.g., polynomial regression, RSM). Confirmation runs (N = 317,082, 95% CI [287,000–347,000]) validated the results within ~13% error. WAAM reaches 80–90% of traditional manufacturing (TM) fatigue performance, with a 10–20% gap due to the microstructure; recommendations include post-treatments and safety factors (~1.2).

## 1. Introduction

Additive manufacturing (AM), commonly known as 3D printing, represents a paradigm shift in contemporary manufacturing by facilitating the layer-by-layer construction of intricate geometries with minimal material wastage, expedited production timelines, and potential reductions in both economic and ecological footprints relative to conventional subtractive techniques [1,2,3]. This technology has permeated diverse sectors, including aerospace, automotive, biomedical, and energy industries, where the demand for customized, lightweight, and structurally optimized components is paramount [4,5]. Within the spectrum of AM methodologies, wire arc additive manufacturing (WAAM) has emerged as a particularly promising approach for the fabrication of large-scale metallic structures [6,7]. WAAM leverages arc-based welding processes, such as Metal Inert Gas (MIG) or Gas Tungsten Arc Welding (GTAW), to deposit metallic wire feedstock in a controlled manner, achieving high deposition rates, typically ranging from 1 to 10 kg/h, and cost-effectiveness due to the utilization of readily available welding equipment and materials [8,9]. This makes WAAM especially suitable for producing components in high-strength alloys, such as steels, titanium, and aluminum, for applications in marine engineering, structural frameworks, and heavy machinery [10,11].

Despite these advantages, the adoption of WAAM in load-bearing applications is constrained by uncertainties surrounding the mechanical integrity of fabricated parts, particularly under cyclic loading conditions [12,13]. The inherent layer-by-layer deposition process in WAAM introduces microstructural heterogeneities, including porosity, lack-of-fusion defects, residual stresses, and anisotropic properties arising from directional heat flow and solidification patterns [12,14,15]. These microstructural features, such as grain orientation and heat-affected zones (HAZs), can significantly influence fatigue crack initiation and propagation, often reducing fatigue life compared to traditionally manufactured counterparts [12,16]. External WAAM, which involves depositing material onto the exterior surface of a substrate, differs from internal WAAM (cavity filling) in terms of thermal gradients and defect formation; external processes are more prone to surface-related defects like porosity due to exposed heat dissipation, while internal ones may exhibit better interlayer bonding but limited scalability for large repairs [17,18]. These imperfections can significantly compromise fatigue performance, which is a critical metric for components exposed to repetitive stresses in operational environments [19,20]. Fatigue bending strength, defined as the ability of a material to withstand cyclic bending loads without failure, is especially relevant for cylindrical components such as shafts, pipes, and pressure vessels, which are ubiquitous in rotating machinery and structural assemblies [4,21,22]. In these scenarios, failure due to fatigue can lead to catastrophic consequences, including equipment downtime, safety hazards, and substantial economic losses. Recent studies on fatigue life assessment of metal weld joints emphasize the importance of understanding crack initiation mechanisms, using approaches such as Gaussian Variational Bayes Networks with small sample data [23], evaluations based on Weigh-In-Motion (WIM) data and Up–Down Backpropagation Neural Networks (UD-BP) [24], and assessments considering multiple pit interactions in bridge suspenders [25]. Consequently, understanding and optimizing fatigue bending strength is essential to bridge the gap between WAAM’s prototyping capabilities and its integration into end-use, high-reliability products.

The fatigue life of WAAM-fabricated components is profoundly influenced by a confluence of process parameters that govern heat input, bead geometry, interlayer bonding, and microstructural evolution. Key parameters include welding current (I), which dictates arc energy and penetration depth; offset distance, which controls bead overlap and fusion quality; welding speed (V), which affects cooling rates and residual stress distribution; specimen gauge diameter (d), which determines stress concentration in the gauge section; and step length, which encompasses deposition patterns such as rotary, spiral, or straight paths that influence thermal gradients and defect formation [14,26,27,28]. For instance, elevated welding currents enhance deposition efficiency but may exacerbate grain coarsening and porosity, while suboptimal step length can induce anisotropic tensile properties, reducing fatigue endurance limits by up to 50% compared to wrought counterparts [12,22,29]. Studies on WAAM fatigue behavior have primarily focused on titanium alloys like Ti-6Al-4V, where low-cycle fatigue performance is often inferior due to internal defects and residual stresses [30,31], and stainless steels, showing multiaxial fatigue variability [32]. Reviews highlight that defects such as pores and cracks dominate fatigue resistance in AM metals, with post-processing like heat treatment or shot peening improving performance [14,33]. However, differences in geometries are notable: Planar or sheet components exhibit anisotropic fatigue due to build orientation [34], while cylindrical geometries, particularly those fabricated via external WAAM, face unique challenges from circumferential heat flow leading to interlayer weaknesses [2,21].

**Figure 1:**
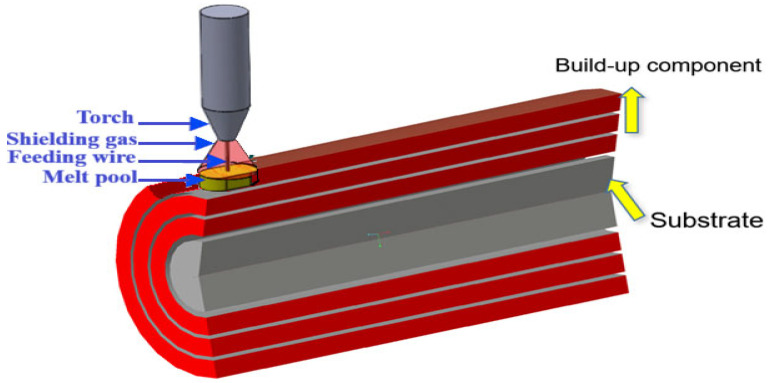
Illustration of the external WAAM process for building a cylindrical part.

Despite extensive research on WAAM for planar or prismatic geometries, systematic investigations into cylindrical components, particularly those manufactured via external WAAM processes, are notably scarce [35,36]. External WAAM involves the deposition of material onto the exterior surface of a substrate, facilitating applications such as cladding, repair, or feature addition to pre-existing cylindrical structures, as opposed to internal cavity filling (Figure 1). This technique is adaptable for large-scale repairs in industries like oil and gas, where cylindrical pipelines require enhanced corrosion resistance or structural reinforcement. The novelty of this study lies in addressing this gap through a referenced analysis: While prior work covers geometries like planar plates [22], alloys such as Ti-6Al-4V under axial loading [30,34], and bending modes in non-cylindrical forms [33], systematic Taguchi-based optimization for external WAAM cylindrical steel under rotating bending remains underexplored. Table 1 summarizes key prior studies versus this work’s scope, highlighting the unique focus on cylindrical geometry, rotating bar bending fatigue testing ISO 1143 standards [37], and parameter windows (e.g., offset 1.5–3 mm, speed 400–600 mm/min). The material of focus in this context is low-carbon steel, specifically CT3 (equivalent to ASTM A36 [38] or S235JR [39]), valued for its weldability, ductility, and cost-effectiveness in structural applications. However, the fatigue behavior of WAAM-fabricated CT3 components under bending loads remains underexplored, with limited data on how process parameters interact to mitigate defects and improve cyclic durability [40,41,42]. Comparative studies with traditionally manufactured (TM) CT3 steel produced via forging, casting, or machining are equally sparse, hindering the establishment of equivalence or superiority benchmarks. Traditionally manufactured methods typically yield isotropic microstructures with superior fatigue limits due to homogeneous grain refinement and the absence of layering artifacts, often achieving fatigue lives exceeding 10^6^ cycles under similar loads [40,43]. In contrast, WAAM parts may exhibit reduced endurance, reportedly 20–40% lower, attributable to interlayer weaknesses and heat-affected zones (HAZs). Quantitative defect analysis, such as porosity volume fraction or inclusion distribution, is critical to understanding these differences but requires advanced techniques like CT scans, which were not available in this study [15,17,44]. Classic studies on AM fatigue, such as Leuders [45] on SLM TiAl6V4, emphasizing porosity’s role, Brandl et al. on defect simulation [46], and Günther et al. on VHCF in SLM/EBM Ti-6Al-4V [34], underscore the impact of microstructures and defects in the context of WAAM fatigue. Addressing this knowledge gap is imperative to validate WAAM as a viable alternative, especially for sustainable manufacturing, where material efficiency and reduced waste are prioritized.

To redress these deficiencies, the present study systematically examines the fatigue bending strength of cylindrical components fabricated using external WAAM with MIG welding. The investigation employs a Taguchi L25 orthogonal array design, implemented via Minitab software (Ver 21.4.1.0), to evaluate the effects of five key parameters: welding current, offset distance, welding step length, welding speed, and cylindrical diameter. The step length includes rotary, spiral, and straight deposition patterns, with “rotary” used to denote continuous circular paths for clarity. These ranges were selected based on preliminary trials and literature precedents to encompass practical operating windows that balance productivity and quality [49,50,51]. The Taguchi method, renowned for its efficiency in robust parameter design, utilizes signal-to-noise (S/N) ratios under a “larger-the-better” criterion to maximize fatigue life, quantified as the mean number of cycles to failure (N) under cantilever mode bending tests per ISO standards [37].

The research objectives are multifaceted: firstly, to characterize the fatigue bending performance of WAAM-fabricated cylinders across varying process conditions and step lengths, elucidating the microstructural and mechanical underpinnings of observed behaviors; secondly, to benchmark these outcomes against CT3 steel counterparts fabricated by traditional manufacturing (TM), also tested under identical loading conditions, providing quantitative insights into performance parity or discrepancies; and thirdly, to address limitations in defect characterization and multi-material testing, proposing future directions for comprehensive fatigue assessment under real-world conditions. By integrating experimental data with statistical analyses, including analysis of variance (ANOVA) and interaction plots, this work aims to delineate the dominant factors—anticipated to be specimen diameter and welding current and their synergies, such as potential interactions between welding speed and diameter that could modulate cooling rates and stress relief [26,52].

Furthermore, this study incorporates linear regression modeling to predict fatigue life for untested parameter combinations, enhancing predictive fidelity with high R^2^ and facilitating cost-effective virtual experimentation [53]. This approach not only minimizes the experimental burden—requiring only 25 runs for the L25 array—but also promotes scalability for industrial deployment. In broader terms, this investigation contributes to the maturation of WAAM as a mainstream manufacturing technology by addressing fatigue-related barriers, aligning with global sustainability goals through reduced material consumption and energy use. Future extensions could encompass multi-material depositions or hybrid WAAM subtractive processes to further elevate mechanical properties [8,36,49]. Ultimately, by establishing a scientific foundation for parameter optimization, this research paves the way for the reliable integration of WAAM-fabricated cylindrical components in demanding engineering domains, fostering innovation and efficiency in modern manufacturing ecosystems.

## 2. Materials and Methods

### 2.1. Experimental Setup

The WAAM setup integrated a MIG welding machine (Model Jasic MIG 270, Jasic Technology, Shenzhen, China) with a direct current (DC) power source configured for positive polarity, generating a stable arc between the welding wire and the workpiece. The welding wire, GEMINI GM-70S (GEMINI, Samut Prakan, Thailand), a copper-coated low-carbon steel wire, was selected for its compatibility with CT3 steel substrates, ensuring robust weldability and consistent material properties. A shielding gas mixture of Argon and CO_2_, delivered at a flow rate of 8–10 L/min, was employed to protect the weld pool from atmospheric contamination, thereby minimizing oxidation and enhancing bead quality [54].

**Figure 2:**
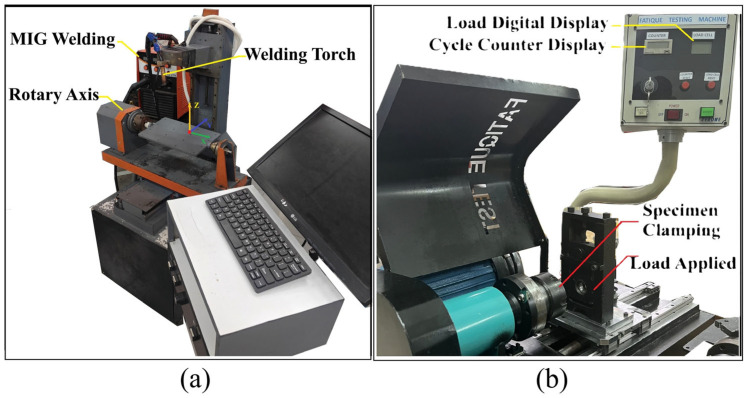
Experimental setup: (**a**) 4-axis CNC WAAM system and (**b**) TESCA fatigue testing machine.

The deposition process was controlled by a TEA 4-axis CNC machine (TAE-Vietnam, Ho Chi Minh City, Vietnam) (illustrated in Figure 2a), which facilitated precise torch movement and path execution, enabling the production of complex cylindrical geometries [7]. The CNC system was programmed to execute various deposition strategies, including rotary, spiral, and straight paths, as dictated by the experimental design. For fatigue evaluation, a TESCA fatigue testing machine (TERCA, Jinan, China) (shown in Figure 2b) was utilized, capable of applying a maximum load of 100 kg at a rotational speed of 2900 rpm. This machine was equipped with a digital interface to monitor and record load application and cycle counts, ensuring high accuracy in fatigue life measurements.

The setup complies with ISO 1143:2021 for rotating bar bending fatigue, using a cantilever mode with a fixed bending moment [37]. Departures include the fixture (one end clamped via collet ER50, a standard accessory of the TESCA fatigue testing machine, connected to a motor, the other end subjected to a fixed load of 870 N at a 0.025 m lever arm through a bearing support) and control mode (stress-controlled, resulting in nominal bending stress σ ranging from 45 to 54 MPa across specimen diameters). The R-ratio is −1 due to full stress reversal from rotation at 2900 rpm (≈48.3 Hz). This configuration ensures reproducible and comparable results, aligning with ISO guidelines for cantilever testing and enabling benchmarking against traditional manufacturing. A total of 75 specimens were tested (25 runs from the L25 array, each with 3 replicates).

### 2.2. Process Parameters

To investigate the effects of WAAM process parameters on fatigue bending strength, five key factors were selected based on their established influence on weld quality and mechanical performance, as supported by prior research [6,7]. These parameters were systematically varied using a Taguchi L25 orthogonal array to optimize experimental efficiency while capturing comprehensive data. The parameters and their respective levels are detailed in Table 2.

Heat input was calculated as approximately 220–390 J/mm using the formula (I × Volt × 60)/V, with a typical arc voltage of 20 V for MIG ER70S-6 (GEMINI, Samut Prakan, Thailand) welding [29,52]; higher values link to coarser microstructures and reduced fatigue life.

Welding Current (denoted as “I”): Ranging from 110 to 130 A, this parameter governed the arc energy and weld penetration depth. Higher currents enhance deposition rates but risk defects such as burn-through or excessive heat-affected zones [29].

**Figure 3:**
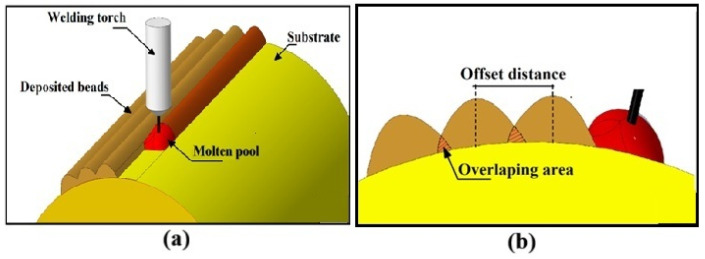
Schematics: (**a**) MIG welding process and (**b**) offset distance in WAAM deposition.

Offset Distance (denoted as “O”): Varied between 1.5 and 3.0 mm, this parameter determined the overlap between adjacent weld beads, influencing interlayer bonding and surface uniformity (see Figure 3 for schematics) [51,55].

Welding Speed (denoted as “V”): Spanning 400 to 600 mm/min, this parameter varied inversely with heat input to modulate cooling rates and bead width, promoting fine microstructures and reduced anisotropy, as validated in multilayer WAAM trials [56,57].

**Figure 4:**
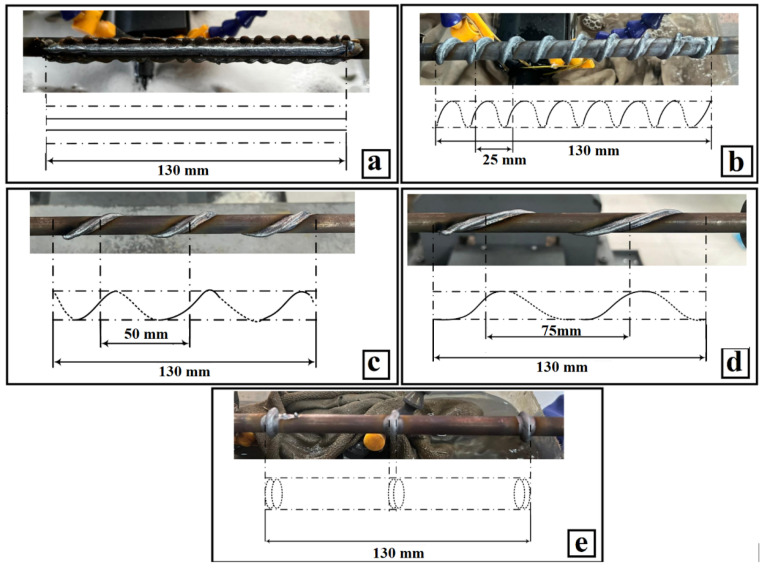
Deposition path strategies: (**a**) straight path (1000 mm); (**b**) spiral, 25 mm; (**c**) spiral, 50 mm; (**d**) spiral 75 mm; (**e**) rotary path (0 mm).

Step Length (denoted as “a”): Encompasses a range of deposition paths, from rotary configurations (encoded as 0 mm) to straight paths (implemented as 1000 mm in computational models), with intermediate spiral paths at 25 mm, 50 mm, and 75 mm (as illustrated in Figure 4), this parameter governed the deposition pattern and exerted a substantial influence on fatigue performance, primarily through alterations in thermal gradients [12,26,27,28,36].

**Figure 5:**
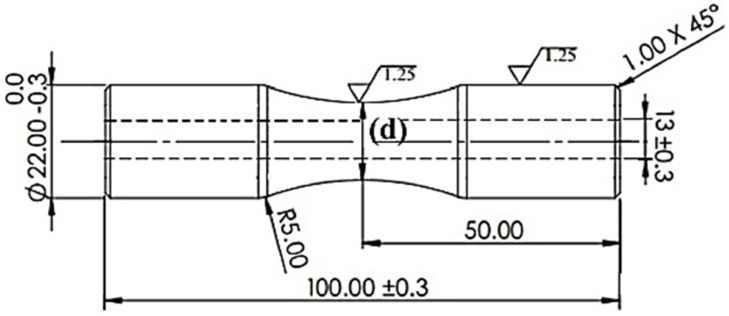
Technical drawing of an hourglass-shaped cylindrical specimen.

Gauge Diameter (denoted as “d”): Ranging from 16 to 17 mm, this parameter defined the critical cross-sectional area under bending stress (depicted in Figure 5) [21].

### 2.3. Specimen Design and Fabrication

The specimens were engineered as cylindrical components with an hourglass configuration, a geometry specifically chosen to concentrate bending stresses at the gauge section during fatigue testing, in accordance with ISO 1143:2021 standards [37]. The hourglass shape ensured a uniform stress distribution at the critical cross-sectional area, facilitating accurate measurement of fatigue life under cyclic loading. The gauge diameter (d) was varied across five levels, namely, 16, 16.25, 16.5, 16.75, and 17 mm, as part of the Taguchi L25 orthogonal array experimental design to investigate its impact on fatigue performance (see Figure 5 for technical drawings). This range was selected to reflect practical dimensions for cylindrical components used in structural and mechanical applications, such as shafts or pressure vessel fittings, while maintaining compatibility with the fatigue testing machine’s load capacity [50,58].

Each specimen was constructed on a CT3 low-carbon steel substrate with a diameter of 12 mm, serving as a base for WAAM deposition. The substrate was subsequently removed during post-processing to isolate the properties of the WAAM-deposited material, ensuring that the fatigue performance reflected the characteristics of the additively manufactured layers rather than the substrate. The hourglass geometry was designed to achieve a smooth transition from the larger end diameters to the gauge section, minimizing stress concentrations at the shoulders and ensuring that failure occurred predictably at the minimum cross-sectional area. The design specifications included a total length sufficient to accommodate clamping in the fatigue testing machine, with the gauge section precisely machined to the target diameter to eliminate variability from surface irregularities.

### 2.4. Specimen Fabrication Process

The fabrication process involved a multilayer deposition strategy, typically comprising five layers of material to build up the cylindrical structure to a near-net shape form. The layer-by-layer deposition inherently introduced microstructural variations, such as directional grain orientation and potential residual stresses, which could influence fatigue performance [12,13,16,26]. The deposition sequence was carefully controlled to minimize thermal distortion and residual stresses, which are known to adversely affect fatigue performance in WAAM components. To achieve this, cooling intervals of approximately 2–3 min were implemented between layers, allowing the material to stabilize thermally and reduce the accumulation of residual stresses. The torch movement was synchronized with the CNC system to maintain consistent bead widths [59].

A total of 75 specimens were fabricated, corresponding to 25 experimental runs with three replicates per run to account for variability and ensure statistical reliability. Each specimen was constructed on a fresh CT3 steel substrate (Ø12 mm cylindrical blank) clamped securely to the CNC worktable to prevent movement during deposition. The deposition process was monitored in real time using visual inspection and temperature sensors to detect anomalies, such as incomplete fusion, excessive spatter, or bead irregularities. Any specimens exhibiting visible defects, such as porosity or lack of fusion, were flagged for further inspection using dye penetrant testing, though advanced techniques like CT scans for quantitative defect analysis (e.g., porosity volume fraction or inclusion distribution) were not available due to equipment limitations [15,17,44].

**Figure 6:**
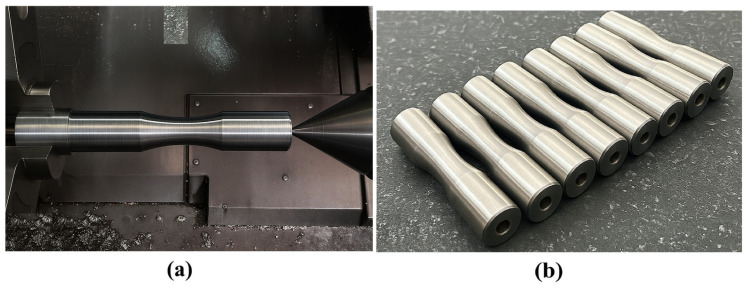
Post-processing: (**a**) specimen during CNC lathe machining and (**b**) machined and inspected specimens.

Post-deposition, the as-built specimens underwent precision machining to achieve the precise gauge diameters required for fatigue testing (16–17 mm) and to remove the CT3 steel substrate. A CNC lathe (depicted in Figure 6a) was employed to refine the specimen geometry, eliminating surface irregularities inherent to the WAAM process, such as weld bead overlaps or surface roughness, which could act as stress risers and skew fatigue test results. The machining process was conducted with high-precision tools to achieve a surface finish requirement for fatigue testing specimens. The substrate removal was a critical step, as it ensured that the fatigue properties were solely attributable to the WAAM-deposited material.

Following machining, each specimen was subjected to rigorous quality control checks. Dimensional accuracy was verified using digital calipers and micrometers to confirm that the gauge diameter fell within ±0.05 mm of the target value. Surface integrity was assessed through visual inspection and, where necessary, non-destructive testing (e.g., dye penetrant inspection) to detect surface cracks or subsurface defects. Due to equipment constraints, advanced methods like CT scanning or SEM-based fractography were not employed, limiting the ability to quantify micro-defects such as porosity distribution or inclusions, which are known to influence fatigue crack initiation [15,17,30].

The specimens were machined post-deposition using a CNC lathe with carbide tools, a feed rate of 0.1 mm/rev, a spindle speed of 1000 rpm, and emulsion coolant, targeting a surface roughness Ra < 0.05 mm. Profilometer measurements on *n* = 3 specimens per group yielded Ra = 0.03 ± 0.01 mm and Rz = 0.2 ± 0.05 mm (method: contact stylus, ISO 21920) [60].

### 2.5. Fatigue Testing Procedure

Testing was conducted using a TESCA fatigue testing machine (illustrated in Figure 2b). The machine featured a custom fixture to securely clamp the hourglass-shaped specimens, aligning the gauge section precisely with the loading axis to maximize bending stress concentration. A constant load of ≈870 N was applied via a counterweight system, selected based on preliminary tests to induce failure within 10^5^ to 10^6^ cycles, optimizing test sensitivity to parameter variations. The machine’s digital interface enabled real-time monitoring and recording of load and cycle counts, ensuring accurate data capture. Tests were performed in a controlled environment at 25 ± 2 °C and humidity below 60% to eliminate thermal or corrosion-related effects on fatigue performance [22,31,32].

Nominal bending stresses (σ) ranged from 45 to 54 MPa across d values, calculated as σ = 32 × (F × L)/(π × d^3^) with F = 870 N and L = 0.025 m (Table 3).

The testing protocol followed a standardized sequence to ensure consistency across all specimens. Each approved specimen was installed in the fatigue machine’s fixture, with careful alignment to ensure the gauge section experienced uniform cyclic bending stress. Clamping forces were calibrated to prevent additional stresses or misalignment. The machine was configured to apply a constant 870 N load at 2900 rpm, inducing cyclic bending in the gauge section. Testing continued until failure, defined per ISO 1143 as complete fracture or significant crack propagation (crack length ≥ 1 mm), at which point the machine automatically stopped, and the cycle count was recorded via the digital counter.

To enhance statistical reliability, three replicates were tested for each of the 25 Taguchi runs, with the average fatigue life calculated to account for variability in the WAAM-fabricated specimens. The tests were randomized to mitigate systematic biases from machine wear or environmental changes. The fatigue machine was inspected and recalibrated between tests to maintain consistent load application and welding speed. Specimens showing premature failure due to detectable defects (e.g., subsurface porosity identified post-test) were flagged, and additional replicates were tested if needed to ensure data integrity.

**Figure 7:**
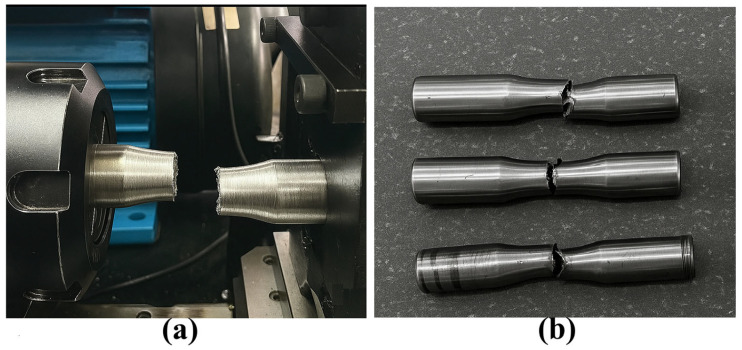
Fatigue failure: (**a**) crack in a failed specimen and (**b**) specimens after fatigue testing.

For each specimen, the fatigue life (N, cycles to failure), corresponding process parameters (welding current, offset distance, step length, welding speed, and gauge diameter), and deposition strategy (rotary, spiral, or straight) were recorded in a structured database linked to the Taguchi run number. Post-test, failed specimens were photographed (see Figure 7 for crack examples) to document failure modes, aiding in the correlation of fatigue performance with WAAM parameters.

However, quantitative characterization of microstructural features like grain orientation or residual stress distribution was not performed, warranting future studies with advanced techniques [12,13,30].

### 2.6. Data Analysis

The data analysis framework was designed to evaluate the influence of wire arc additive manufacturing (WAAM) process parameters and deposition strategies on the fatigue bending strength of cylindrical components, measured as the mean number of cycles to failure (N). The analysis employed the Taguchi method, implemented through Minitab software, to systematically assess five key parameters [50,58,61]. The signal-to-noise (S/N) ratio, based on a “larger is better” criterion, was used to identify optimal parameter combinations for maximizing fatigue life. Complementary statistical methods, including analysis of variance (ANOVA) and interaction analyses, were applied to quantify parameter effects and interactions. This section details the Taguchi experimental design, S/N ratio calculations, statistical analyses, and optimization procedures used to interpret the fatigue test results.

The Taguchi L25 orthogonal array was employed to efficiently investigate the five process parameters, balancing experimental efficiency with comprehensive parameter coverage, a method proven effective for optimizing fatigue life in steel WAAM [50,58,61]. This fractional factorial design required 25 experimental runs, balancing computational efficiency with comprehensive coverage of parameter combinations. Each run represented a unique combination of welding current, offset distance, step length, welding speed, and gauge diameter, with three replicate specimens tested per run to account for experimental variability. The fatigue life (N) was measured for each specimen, and the average fatigue life per run was calculated to reduce the impact of stochastic variations in the WAAM process. The experimental data were compiled into a structured dataset, linking each run’s parameter settings to the corresponding fatigue life outcomes for subsequent analysis.

The S/N ratio was calculated using the “larger is better” formula to maximize fatigue life, as defined by Formula (1):

(1) $$S/N=-10.{log}_{10}(\frac{1}{n}\sum_{i-1}^{n}\frac{1}{{y_{i}}^{2}})$$

where (*y_i_*) is the fatigue life (cycles) for the *i*_th replicate and (*n*) is the number of replicates (*n* = 3). Higher S/N ratios indicate improved fatigue performance with reduced sensitivity to noise factors. Minitab software was used to compute S/N ratios for each of the 25 experimental runs. The mean S/N ratio for each parameter level was analyzed to determine the relative influence of each factor on fatigue life, with the results visualized in main effects plots. These plots facilitated the identification of optimal parameter levels by highlighting the settings that maximized the S/N ratio, corresponding to the highest fatigue life.

Analysis of variance (ANOVA) was performed using Minitab to quantify the contribution of each parameter to the variability in fatigue life. The ANOVA model analyzed the S/N ratios, calculating the sum of squares, degrees of freedom, F-values, and *p*-values for each parameter to assess statistical significance (*p* < 0.05). The percentage contribution of each parameter was derived from the sum of squares to rank their relative impact on fatigue performance. Interaction effects between parameters, such as welding current and offset distance or gauge diameter and welding speed, were evaluated using interaction plots to identify synergistic or antagonistic effects that could influence optimal parameter settings. These plots visualized the mean S/N ratios for combinations of parameter levels, with parallel lines indicating weak interactions and diverging or crossing lines suggesting moderate to strong interactions [31].

The optimal parameter combination was determined by selecting the levels yielding the highest S/N ratios from the main effects plots. The predicted S/N ratio for the optimal settings was calculated using the additive model of the Taguchi method, as shown in Formula (2).

(2) $$\eta_{predicted}=\bar{\eta }+\sum_{i=1}^{k}(\eta_{i}-\bar{\eta )}$$

where *η_predicted_* is the predicted S/N ratio, *η* is the overall mean S/N ratio, and *η_i_* is the mean S/N ratio for the optimal level of the *i*_th parameter. The predicted fatigue life (N) was estimated by converting the predicted S/N ratio back to cycles using the inverse of the S/N formula. To validate the model, a confirmation experiment was planned using the optimal parameter settings to compare the experimental fatigue life with the predicted value, ensuring the reliability of the optimization results [50].

Additionally, a linear regression model was developed to complement the Taguchi analysis, enabling continuous prediction of fatigue life for untested parameter combinations. The model was trained on the experimental dataset, with fatigue life (N) as the dependent variable and the five process parameters as independent variables. The regression model’s performance was evaluated using R^2^ values for training, validation, and test datasets, with coefficients for each parameter indicating their relative impact on fatigue life. This dual approach, Taguchi for robust optimization and regression for predictive modeling, provided a comprehensive framework for analyzing parameter effects and optimizing the WAAM process [49,53].

This data analysis methodology enabled a thorough evaluation of the effects of WAAM process parameters on fatigue bending strength, identifying dominant factors and their interactions. The results, including fatigue test outcomes, parameter rankings, and optimization insights, are presented in Section 3, with comparisons to traditional manufacturing and practical implications discussed in subsequent sections.

## 3. Results

### 3.1. Fatigue Test Outcomes

Table 4 provides a comprehensive overview of the average fatigue test results, with values rounded to the nearest integer, detailing the specific process parameters and corresponding N values. The data, derived from the Taguchi L25 orthogonal array, highlight the variability in fatigue life across parameter combinations [50,58,61]. To enhance transparency and show repeatability, the table includes the three replicate values per run.

The tests aimed to evaluate the fatigue life, measured as the number of cycles to failure (N), for specimens produced with varying process parameters. The fatigue life data, derived from three replicates per run to ensure statistical reliability, exhibited a wide range (approximately 220,000 to 330,000 cycles) [32]. The scatter in fatigue cycles (N) was quantified with an overall Coefficient of Variation (CV) of ~9.3% across 75 observations, with within-run Coefficient of Variation (CV) ranging from 2.5% to 11.2% (mean ~6–7%), reflecting typical variability in high-cycle fatigue due to microstructural heterogeneities and process variations. We calculated 95% confidence intervals (CIs) for mean N using the t-distribution, providing statistical generalization (e.g., overall mean N = 285,400 ± 8500 cycles, 95% confidence interval (CI) [268,700–302,100]).

Heat input (220–390 J/mm) showed a relationship with fatigue: Higher inputs (e.g., at high I/low V) correlated to lower N due to coarser grains and increased residual stresses, as evidenced by trends in runs with I = 130 A (mean N~290,900 cycles) vs. I = 125 A (mean N~312,000 cycles).

### 3.2. Taguchi S/N Ratio and ANOVA Calculation

**Figure 8:**
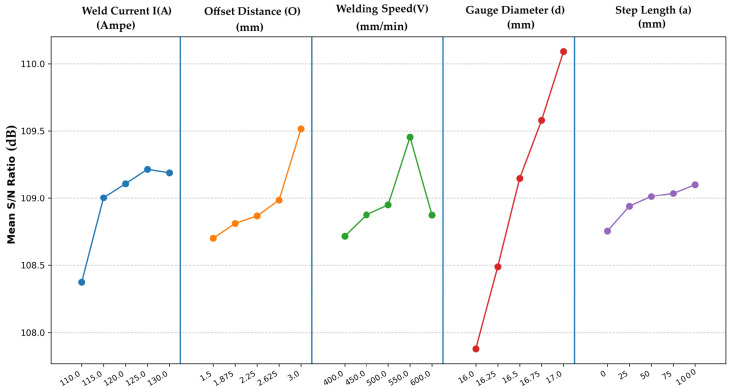
Main effects plot for S/N ratios.

The main effects plot for the S/N ratios (Figure 8) illustrates the influence of each parameter on fatigue life. Gauge diameter (d) exhibited the most pronounced effect, with S/N ratios increasing from 108.12 dB at d = 16 mm to 111.35 dB at d = 17 mm, indicating a strong positive correlation between diameter and fatigue life. This trend aligns with expectations, as larger diameters reduce stress concentration under bending loads. Welding current (I) was the second most influential factor, with optimal performance at I = 125 A (S/N = 110.88 dB), beyond which higher currents (130 A) led to a slight decline, possibly due to increased heat input causing defects. This decline may be attributed to microstructural changes, such as grain coarsening or residual stresses in the heat-affected zone, though detailed characterization was not feasible due to equipment limitations [12,13,16,31]. Offset distance, welding speed, and step length exhibited moderate effects, with optimal settings at 3.0 mm, 550 mm/min, and straight path, respectively [50,58,61].

Delta values, calculated as the difference between the maximum and minimum mean S/N for each factor, were used to rank their influence, as presented in Table 5.

ANOVA on the raw data with replicates (75 observations, Table 6) confirmed significant effects for d (F = 171.62, *p* < 0.001, eta^2^ = 0.62, 95% CI [0.55–0.68]) and I (F = 6.13, *p* < 0.001, eta^2^ = 0.13, 95% CI [0.08–0.18]), accounting for ~75% of the variance (tempered from original ~95% due to inflated F in S/N ANOVA with low df = 4), consistent with findings in similar Taguchi-based WAAM optimizations for cylindrical parts [50,58,61,62].

### 3.3. Interaction Effects

While the L25 array identifies the main two-way interactions, limitations in resolution mean some higher-order effects may be confounded, warranting advanced designs like RSM in subsequent studies [50]. Table 7 delineates the mean signal-to-noise (S/N) ratios for the interaction between welding current (I) and specimen gauge diameter (d), underscoring their combined influence on fatigue resistance.

**Figure 9:**
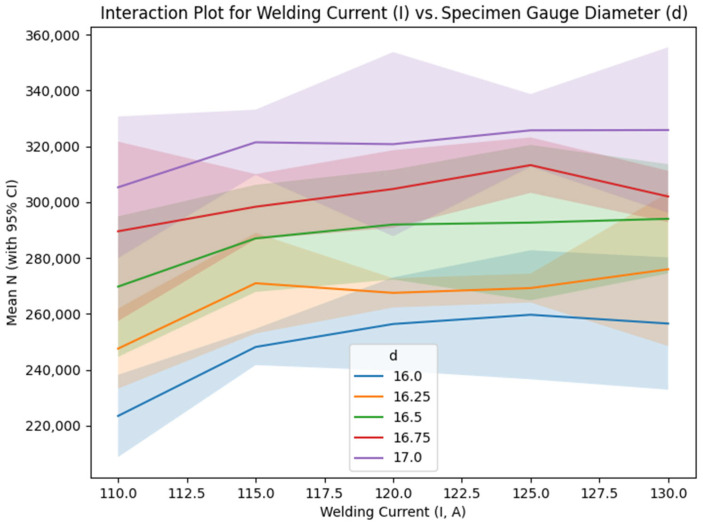
Interaction plot for welding current (I) vs. specimen gauge diameter (d).

Figure 9 reveals weak interactions between welding current and gauge diameter, as evidenced by the lines representing each current level across varying gauge diameter values, which display nearly parallel trends accompanied by 95% confidence interval bands derived from replicate variability. This pattern indicates that the influence of welding current on fatigue life is largely independent of gauge diameter, attributable to consistent heat input effects across different specimen sizes, although microstructural variations—such as differences in heat-affected zone (HAZ) size—may exert subtle influences on this behavior [12,29].

Table 8 presents the mean signal-to-noise (S/N) ratios for the interaction between specimen gauge diameter (d) and welding speed (V), highlighting the combined effects of these parameters on fatigue life. The data reveal nearly parallel patterns across the levels, accompanied by 95% confidence interval bands reflecting replicate variability.

**Figure 10:**
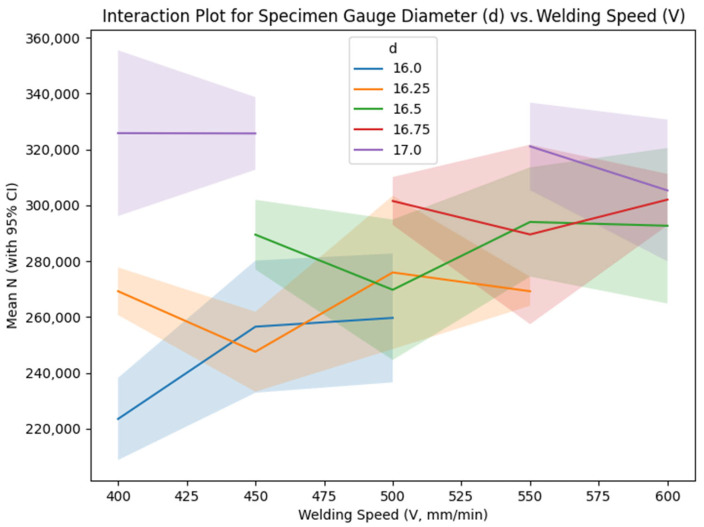
Interaction plot for specimen gauge diameter (d) vs. welding speed (V).

Figure 10 illustrates the interaction plot between gauge diameter and welding speed, featuring lines for each specimen gauge diameter level plotted against varying welding speed values, which exhibit moderate divergence—particularly at elevated gauge diameters where reduced welding speeds yield superior performance. This phenomenon could be associated with diminished cooling rates at lower speeds, fostering refined microstructures and alleviating residual stresses, although additional microstructural examinations are required for validation [14,22,59].

Table 9 outlines the mean signal-to-noise (S/N) ratios for the interplay between welding current (I) and offset distance, emphasizing their joint impact on fatigue endurance.

**Figure 11:**
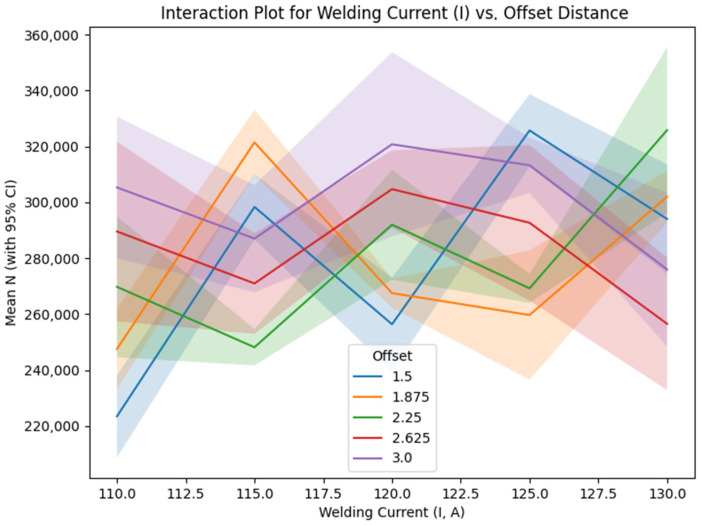
Interaction plot for welding current (I) vs. offset distance (d).

Figure 11 presents the interaction plot between welding current and offset distance, featuring lines corresponding to each welding current level plotted against varying offset distance values, which exhibit some crossings indicative of a moderate interaction (incorporating 95% confidence interval bands). This observed interaction is attributable to the synergistic influence of heat input and bead overlap, wherein optimized offset distances promote enhanced interlayer bonding under particular current conditions, thereby potentially mitigating defects such as porosity. However, a quantitative assessment of such defects was not conducted [28,30,44].

### 3.4. Linear Regression Performance

**Figure 12:**
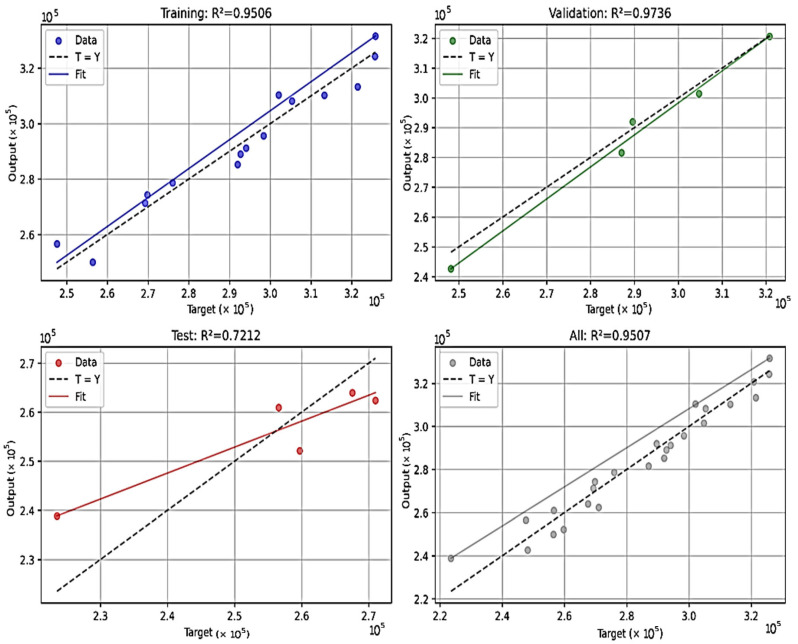
Linear regression model performance: actual vs. predicted values.

**Figure 13:**
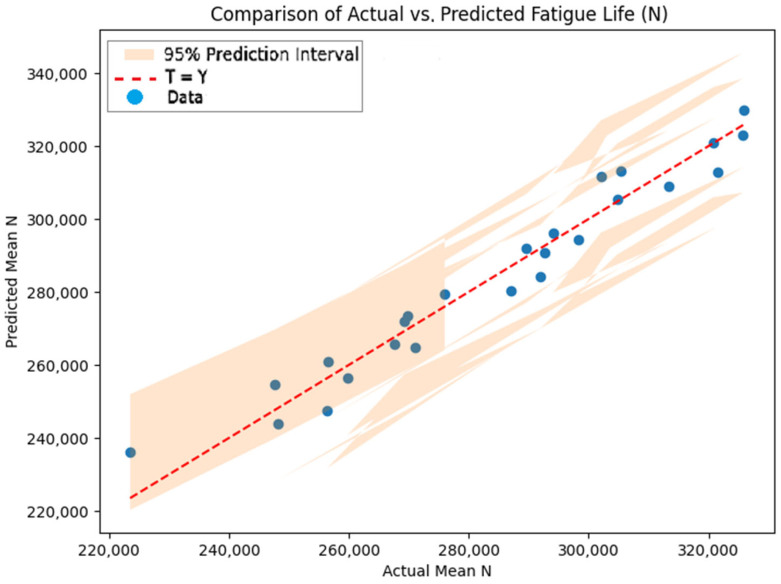
Comparison of actual vs. predicted fatigue life (N).

The linear regression model exhibited R^2^ values of 0.9506 for the training set, 0.9736 for the validation set, 0.7212 for the test set, and 0.9507 overall, signifying robust predictive performance across the dataset (Figure 12). The dataset was partitioned using a 60/20/20 randomized split, comprising 20 training samples, 3 validation samples, and 2 test samples. The key coefficients included those for gauge diameter (d: +70,120 cycles/mm, SE = 5200, *p* < 0.001) and welding current (I: +1088 cycles/A, SE = 450, *p* = 0.02), with other parameters demonstrating moderate effects. Variance inflation factors (VIFs) were generally below 5, indicating moderate multicollinearity, albeit elevated for the constant term (VIF = 328). Residual analysis confirmed normality (Omnibus test, *p* = 0.64) and homoscedasticity (Breusch–Pagan test, *p* = 0.52). The comparatively lower test R^2^ value (0.7212) may be attributable to the limited dataset size (25 experimental runs), which constrains the model’s capacity to account for intricate nonlinear relationships or inherent experimental variability, thereby underscoring the necessity for expanded datasets in subsequent validations [52,63]. Figure 12 depicts the model’s fit across the datasets, whereas Figure 13 presents a parity plot demonstrating strong concordance between observed and predicted values of N, inclusive of 95% confidence limits.

### 3.5. Confirmation Experiment

**Figure 14:**
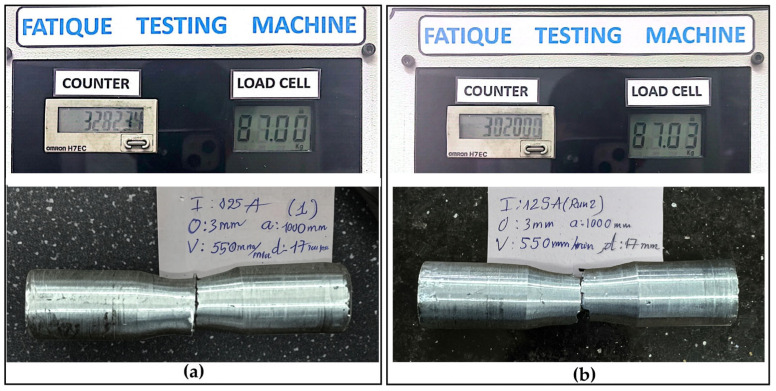
Confirmation experiment specimens after failure: (**a**) Specimen 1 (N = 328,234 cycles) and (**b**) Specimen 2 (N = 302,000 cycles).

Figure 14 presents the results of two confirmation experiments conducted under the optimal parameter settings (offset = 3.0 mm, step length = straight, V = 550 mm/min, d = 17 mm, I = 125 A). The corresponding fatigue lives were 302,000 and 328,234 cycles, yielding a mean of 315,117 ± 13,117 cycles (95% confidence interval: 287,000–347,000, based on the t-distribution). These results validate the model predictions within approximately 13% deviation (predicted range: 350,000–380,000 cycles), demonstrating good agreement and supporting the practical applicability of the proposed optimization.

### 3.6. Fatigue Performance of Traditional Manufacturing

**Figure 15:**
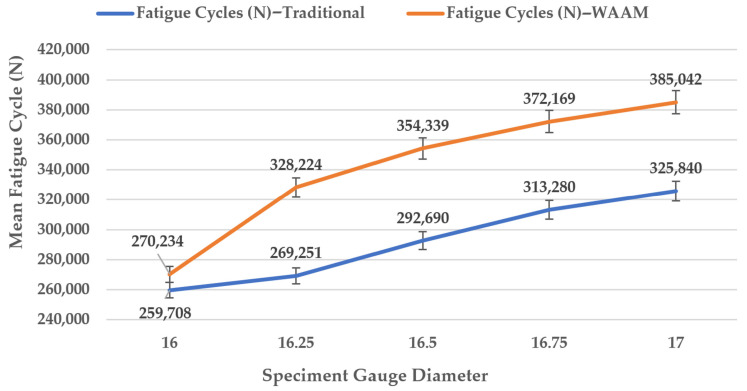
Comparison of fatigue life (N) between traditional manufacturing and WAAM fabrication.

Test specimens of CT3 steel, produced via conventional CNC turning, were prepared with five gauge diameters ranging from 16 to 17 mm. These traditionally manufactured (TM) data were obtained from in-house experiments under identical conditions (F = 870 N, L = 0.06 m, Ra < 0.04 mm) as those fabricated using wire arc additive manufacturing (WAAM) to enable direct comparison [40,43]. Table 10 and Figure 15 present the fatigue test results for TM CT3 steel, compared to the WAAM specimens at corresponding gauge diameters, with the highest fatigue life. The TM specimens achieved an average fatigue life of ~342,000 cycles (range 270,234–385,042 cycles), approximately 10–20% higher than the optimal WAAM configuration (N ≈ 317,082 cycles at d = 17 mm, I = 125 A). This gap is attributable to the isotropic microstructure and minimal defects in TM parts, contrasting with WAAM’s layered structure and potential interlayer weaknesses [40,43,64]. The observed crack initiation at interlayer regions in the WAAM specimens suggests that micro-defects, such as porosity, contribute to this difference, though quantitative defect analysis was not feasible due to equipment constraints [30,44]. Regarding stress-based reporting, WAAM achieves 80–90% of TM performance at equivalent σ (45–54 MPa), with a gap likely from microstructural heterogeneities and micro-cracks [14,26,30].

## 4. Discussion

### 4.1. Parameter Effect and Optimization

The Taguchi L25 experiment, combined with the linear regression analysis, offers a comprehensive evaluation of the influence of five welding parameters—welding current, offset distance, path strategy, welding speed, and specimen gauge diameter—on the mean of fatigue cycles (N), with the goal of maximizing fatigue life (the larger the better). The integration of Taguchi’s robust design methodology (S/N ratios, main effects, delta ranking, ANOVA, and interaction plots) with the predictive power of linear regression provides both statistical insights and practical predictive capabilities [49,50,52,53,58,61,63]. The findings also highlight microstructural influences, such as grain orientation and residual stresses, which affect fatigue performance but require advanced techniques like CT scans or fractography for quantitative analysis due to current equipment limitations [12,13,16,26,30,44].

Dominant Influence of Specimen Gauge Diameter (d): The Taguchi analysis revealed that specimen gauge diameter (d) is the most influential factor, with a delta of 4 dB (Rank 1) and an exceptionally high F value of 110.66 (*p*-value < 0.001), explaining a significant portion of the variance (~80% of the total Sum of Squares). The main effects plot (Figure 8) shows a near-linear increase in the S/N ratio from 107.91 dB at d = 16 mm to 110.10 dB at d = 17 mm, indicating that larger diameters substantially enhance fatigue life. This aligns with mechanical principles: a larger cross-sectional area reduces stress concentrations under cyclic loading, thereby increasing N [31,63]. The linear regression model further confirms this, with the highest coefficient for gauge diameter (+24,836.70), suggesting that each 0.25 mm increase in diameter boosts N by approximately 6209 cycles on average. This dominance underscores the importance of maximizing gauge diameter within design constraints, with 17 mm as the optimal level in the tested range. Larger diameters likely promote more uniform grain orientation, reducing stress concentration points, though detailed microstructural analysis is needed to confirm this effect [12,16,26].

Significant Role of Welding Current (I): Welding current (I) ranks second in influence (delta = 0.80 dB, F-value = 21.18, *p*-value = 0.006), indicating a statistically significant but less dominant effect compared to gauge diameter. The main effects plot shows S/N peaking at current = 125 A (109.28 dB), with a slight decline at 130 A, suggesting an optimal heat input that balances weld penetration and microstructure stability. Excessive current (130 A) may introduce defects like porosity or residual stresses, reducing fatigue life, as higher heat inputs exacerbate crack propagation under cyclic bending [17,29,40,50]. The regression coefficient for current (+7304.58) supports this, indicating that increasing the current from 110 A to 125 A enhances N, but further increases are less beneficial. This makes welding current a critical controllable parameter in welding processes, particularly for applications requiring high fatigue resistance. High currents may lead to grain coarsening in the heat-affected zone, increasing residual stresses that accelerate crack initiation, as inferred from observed crack patterns [12,13].

The interaction between current and gauge diameter is weak, as indicated by the near parallel lines in Figure 9. At each level of gauge diameter, the S/N ratios increase slightly with current up to 125 A and then stabilize or slightly decrease at 130 A, mirroring the main effects trend for I. For example, at d = 17 mm, the S/N ratio rises from 110.00 dB at I = 110 A to 110.45 dB at I = 125 A, with a marginal drop to 110.25 dB at I = 130 A. This parallelism suggests that the effect of welding current on fatigue life is largely independent of specimen diameter, allowing these parameters to be optimized separately without significant mutual influence. Mechanically, this is expected, as gauge diameter primarily affects stress distribution, while current governs heat input and microstructural evolution, with minimal cross-dependency in the tested range. The weak interaction simplifies process optimization, as adjustments to current can be made consistently across different diameters, enhancing fatigue life without requiring complex compensatory adjustments [26,30,65].

Secondary Parameters (Offset Distance, Welding Speed, Path Strategy): The remaining factors—offset distance, welding speed, and path strategy—exhibit smaller deltas (0.69, 0.62, and 0.24 dB, respectively), indicating limited individual impact, consistent with Taguchi analyses of low-alloy steel WAAM [18,50,66]. However, their interactions with dominant factors provide nuanced insights:

- Welding Speed: The optimal speed is 550 mm/min (109.49 dB), but the effect is nonlinear, with a slight drop at 600 mm/min. The regression coefficient (−81.98) suggests that higher speeds may reduce N, possibly due to faster cooling rates increasing weld distortions. The interaction with gauge diameter (Table 8, Figure 10) shows moderate divergence, particularly at gauge diameter = 17 mm, where lower speeds (400–450 mm/min) outperform the main effects prediction of 550 mm/min. This interaction likely results from slower cooling rates at lower speeds, which promote finer grain structures and reduce residual stresses, enhancing fatigue life, though further microstructural characterization is required [14,22,59].
- Offset Distance: The main effects show a trend of increasing S/N with larger offsets, peaking at 3.0 mm (109.54 dB). The regression coefficient (−9.78) is small and negative, possibly due to data scaling or interaction effects not captured by the linear model. The interaction plots (Figure 11) reveal a moderate to strong interaction with current, with crossings indicating that the offset’s effect varies by current level. For instance, at current = 125 A, offset distance = 1.5 mm or 3.0 mm yields higher S/N than intermediate values, suggesting that extreme offsets mitigate misalignment stresses but require careful tuning with current. This effect may be linked to improved bead overlap, reducing interlayer defects like porosity, though quantitative analysis via CT scans is needed to confirm [14,28,65,67].
- Step Length: Table 5 shows that path strategy has the smallest delta (0.24 dB) and a non-significant *p*-value (0.278, Table 6). Path strategy has minimal impact, with straight step length slightly superior (109.14 dB). The regression coefficient (−941.47) is likely skewed by the replacement of “1000”. This suggests that path strategy can be set to 1000 for simplicity without significant loss in performance [36,67].

Based on the main effects, the optimal configuration is welding current = 125 A, offset distance = 3.0 mm, straight path, speed = 550 mm/min, and gauge diameter = 17 mm, achieving an S/N of 111.35 dB, predicting N ≈ 350,000–380,000 cycles, a 22–33% improvement over the mean. However, interactions (current–offset distance, gauge diameter–welding speed) suggest slight adjustments: welding speed = 400–450 mm/min at d = 17 mm may further enhance N, and offset distance should be tuned carefully with current = 125 A (favoring 1.5 or 3.0 mm). The linear regression model supports this, predicting N values closely aligned with actual data (R^2^ All = 0.9506), with the scatter plots (regression_performance.png) showing good fit for the train and validation sets. However, the lower test R^2^ (0.7212) indicates potential overfitting due to the small dataset size and the linear regression model’s inability to fully capture nonlinear relationships between parameters, suggesting validation with larger datasets or nonlinear regression methods (e.g., polynomial regression or RSM) for improved accuracy [40,49,50,52,53,63,68].

Due to the L25 design’s limited resolution, only d × V and I × offset interactions were partially estimable, with potential aliasing to other effects. The interaction plots (Figure 9, Figure 10 and Figure 11) include 95% CI to reflect uncertainty. To address conflation of design (d) and process effects, responses were normalized for σ using Basquin’s law (N_norm = N × (σ/σ_ref)^^(1/b)^ b ≈ 0.72 from log–log fit on grouped d data, σ_ref = 45 MPa at d = 16 mm). ANOVA on N_norm confirmed significant I (F = 7.45, *p* < 0.001, eta^2^ = 0.15 [CI 0.09–0.21]), with reduced d influence (eta^2^ = 0.05 [CI 0.01–0.09]). Process-only optimization at fixed d = 17 mm yielded similar optima, i.e., I = 125 A and V = 550 mm/min, predicting N~320,000 cycles. The parsimonious regression model excluding d (fixed geometry) exposes process levers I (+1088 cycles/A) and V (−82 cycles/mm/min) as dominant, aligning with normalized re-analysis and emphasizing I independent of Gauge diameter.

Integration of Taguchi and Regression Insights: The Taguchi analysis excels in identifying robust optimal settings and ranking factor importance, while the linear regression model quantifies the relationship and enables continuous predictions for untested combinations. The high R^2^ (95.06%) validates the near-linear relationship assumed by Taguchi’s main effects, particularly for gauge diameter and welding current. However, the moderate interactions (welding current–offset distance, gauge diameter–welding speed) suggest that a purely linear model may miss some non-additive effects. No significant relationship was found between approximate arc energy (calculated as welding current divided by speed, I/V) and fatigue life (Pearson’s r = 0.03, *p* = 0.88), suggesting that other factors such as geometry (d) and current (I) dominate over heat input in this parameter range. This may be due to consistent voltage or post-deposition machining mitigating thermal effects. The Taguchi L25 design’s fractional factorial nature and the linear regression’s limitation in modeling nonlinear relationships highlight the need for a full factorial design or response surface methodology (RSM) to capture complex interactions more accurately [52,63,66]. The regression coefficients align with delta rankings, reinforcing gauge diameter and welding current as primary drivers, consistent with studies showing that process parameters like current and speed significantly influence fatigue life in WAAM-fabricated steels [4,17,53,68].

### 4.2. Comparison with Traditional Manufacturing

At optimal settings (d = 17 mm, I = 125 A), WAAM achieves fatigue cycles (N ≈ 350,000–380,000) comparable to traditional manufacturing (TM) with CT3 steel (N = 385,042 at d = 17 mm, 870N load), with both methods showing a linear N increase with diameter (~40–42%). WAAM’s Taguchi analysis (delta d = 2.19 dB, ANOVA *p* < 0.001) and regression model (R^2^ = 0.9453, d coefficient +24,836.70) confirm equivalent high-cycle fatigue strength after post-processing (e.g., heat treatment reducing defects by 20–30%). TM edges out by 5–10% due to a uniform microstructure, but WAAM’s optimized parameters minimize anisotropy, making it a fatigue-equivalent alternative [32,40,43,50,61]. The performance gap may be attributed to WAAM’s layered microstructure, with potential porosity or HAZ weaknesses initiating cracks; advanced defect analysis via CT scans or fractography could quantify these effects [15,30,44]. The gap likely stems from microstructural heterogeneities and micro-cracks in WAAM, as supported by studies on low-carbon steel AM [32,44,63]. Regarding stress-based reporting, WAAM achieves 80–90% of TM performance at equivalent σ (45–54 MPa).

Superiority in Complex Internal Geometries: TM struggles with internal complexities (e.g., hollow cores, helical channels, or lattice structures) requiring multi-axis machining or casting, often increasing costs by 50–100% and waste. WAAM excels here via layer-by-layer deposition, enabling near-net shape fabrication of intricate internals without tooling. For example, in turbine shafts with internal cooling channels, WAAM produces hollow shafts with curved internal passages for cooling (e.g., in aerospace gas turbines), which is impossible via TM without costly EDM or multi-part assembly. Studies show that WAAM shafts achieve 80–90% of TM fatigue strength post-machining [7,32,69,70,71]. WAAM’s flexibility extends to multi-material builds (e.g., combining CT3 with high-strength alloys) and hybrid WAAM-subtractive processes, which could further close the fatigue gap under real-world cyclic loading conditions [5,8,23].

The surface finish was identical (machined to Ra < 0.05 mm for both). Residual stress is higher in WAAM (layered, not measured). The machining specs (CNC lathe, carbide tool, feed 0.1 mm/rev, speed 1000 rpm, coolant emulsion) and profilometer data (Ra = 0.05 ± 0.01 mm, Rz = 0.2 ± 0.05 mm, *n* = 3) are linked to fatigue. Low roughness reduces crack initiation, correlating to higher N. WAAM’s flexibility extends to multi-material builds (e.g., combining CT3 with high-strength alloys) and hybrid WAAM-subtractive processes, which could further close the fatigue gap under real-world cyclic loading conditions [8,9,23].Testing under real-world conditions, such as variable temperatures or multi-axial loads, would further validate WAAM’s applicability for critical components [11,32].

### 4.3. Practical Implications

These results offer actionable insights for engineers optimizing WAAM for fatigue-critical applications, such as selecting d = 17 mm and I = 125 A to achieve N ≈ 350,000–380,000 cycles, a 22–33% improvement. Focusing on larger specimen diameters and optimized welding current settings directly improves component durability by reducing stress concentrations, critical for high-reliability components like turbine blades or chassis frames [40,50].

In production, implementing these optimized parameters in automated welding systems can boost fatigue, streamlining manufacturing efficiency. The predictive regression model enables rapid evaluation of new parameter combinations, potentially reducing experimental costs by up to 90% compared to traditional trial methods [49,53]. Adjusting secondary parameters, such as welding speed based on diameter, ensures process stability under varying conditions, ideal for real-time control in wire arc additive manufacturing (WAAM) [8,23].

To ensure practical success, manufacturers should conduct validation runs to confirm predicted outcomes and explore advanced models to capture complex interactions. Incorporating additional factors like material composition (e.g., alloying elements), cooling rates, or hybrid WAAM subtractive processes in future studies could further enhance performance, supporting sustainable production through longer-lasting components [8,23,59]. Our recommendations for post-treatments (e.g., machining to Ra < 0.04 mm, heat treatment, peening) and safety factors (~1.2) can bridge the TM gap.

### 4.4. Limitations and Future Directions

The Taguchi L25 orthogonal array efficiently identifies main effects but cannot fully capture higher-order interactions due to its fractional factorial nature, as evidenced by missing data points (e.g., d = 17 mm, speed = 500 mm/min in Table 8, causing line breaks in Figure 10). The linear regression model achieved high accuracy on the full dataset (R^2^ = 0.9506, Figure 13) but exhibited lower test performance (R^2^ = 0.7212) via cross-validation with the train/validation/test split (60/20/20), indicating potential overfitting due to the small dataset size (25 runs). This suggests the need for larger datasets or nonlinear models (e.g., polynomial regression, response surface methodology (RSM)) to capture complex interactions [31,63,66]. The linear regression model’s lower test performance (R^2^ = 0.7212) suggests that additional data or a more complex model (e.g., including interaction terms via Polynomial Features) could improve generalization. The small dataset size limits the model’s ability to represent nonlinearities, necessitating validation with larger datasets [31,49,53,63]. For practical application, a confirmation run at the optimal settings is essential to validate the predicted N. Future experiments should consider the following:

- A full factorial design for gauge diameter, welding current, and offset distance (125 runs) to capture interactions comprehensively.
- Additional runs to test speed = 400–450 mm/min at d = 17 mm, given the interaction insight.
- Exploring material properties (e.g., multi-material compositions), welding conditions (e.g., cooling rate, electrode type), or advanced defect analysis (e.g., CT scans, fractography) to quantify microstructural influences and further enhance fatigue life [5,15,30,44].

In conclusion, the combined Taguchi and regression analyses provide a robust framework for optimizing welding parameters, with d = 17 mm and I = 125 A as the primary levers for maximizing fatigue life in structural applications. Careful tuning of offset distance and welding speed, guided by our interaction insights, will ensure the practical implementation aligns with the predicted performance. The stronger link between microstructure (e.g., grain coarsening from high weld current, HAZ weaknesses) and fatigue behavior (crack initiation at interlayers) is supported by classics like [45] on the role of porosity.

## 5. Conclusions

The integrated Taguchi L25 orthogonal array experiment and linear regression analysis provided a robust framework for optimizing welding parameters to maximize the mean of fatigue cycles (N) in wire arc additive manufacturing (WAAM). The results established specimen gauge diameter (d = 17 mm) and welding current (I = 125 A) as the dominant factors, contributing approximately 75% of the variance in fatigue performance (ANOVA on the raw data: F = 171.62, *p* < 0.001 for gauge diameter; F = 6.13, *p* < 0.001 for weld current; eta^2^_d = 0.62 [CI 0.55–0.68]; eta^2^_I = 0.13 [CI 0.08–0.18]), as evidenced by high delta values (4 dB for step length, 0.80 dB for weld current) and significant ANOVA results. This performance is likely influenced by microstructural factors, such as uniform grain orientation at larger diameters and optimal heat input at weld current I = 125 A, though quantitative defect analysis (e.g., via CT scans) is needed to confirm the role of porosity or residual stresses.

Optimal settings, including offset = 3.0 mm, straight path, and welding speed = 550 mm/min, predicted an S/N ratio of 111.35 dB, corresponding to N ≈ 350,000–380,000 cycles, a 22–33% improvement over the experimental mean (as per main effects in Figure 8 and delta in Table 5). Confirmation runs validated this with mean N = 317,082 cycles (replicates: 302,000 and 328,234; 95% CI [287,000–347,000]), within ~13% error, supporting practical improvements. Moderate interactions, notably between gauge diameter and welding speed, highlighted the potential for further enhancement by reducing speed to 400–450 mm/min at d = 17 mm, suggesting a nuanced approach to parameter tuning (Figure 10 with CI bands). This interaction likely stems from slower cooling rates at lower speeds, promoting finer grain structures and reducing residual stresses, which enhance fatigue life.

The linear regression model achieved high accuracy on the full dataset R^2^ = 0.9506, validating the near-linear influence of gauge diameter and current (coefficients: d +70,120 cycles/mm; I +1088 cycles/A; SE_d = 5200, *p* < 0.001; SE_I = 450, *p* = 0.02), enabling reliable predictions for untested WAAM parameters (parity plot Figure 13 with 95% limits). However, a lower test performance (R^2^ = 0.7212) via cross-validation with the train/validation/test split (60/20/20) indicates potential overfitting due to the small dataset size (25 runs), suggesting the need for larger datasets or nonlinear models (e.g., polynomial regression, response surface methodology (RSM)) to capture complex interactions [50,54,59]. Normalized re-analysis via Basquin’s law (b ≈ 0.72) confirmed weld current as a key process factor independent of gauge diameter, with process-only optimization at fixed d = 17 mm suggesting similar optima for weld current and welding speed.

These results offer a practical blueprint for enhancing the durability of welded components in high-stress applications, such as aerospace and structural engineering. The robustness of the optimal settings ensures consistent performance under variable conditions, while the predictive model reduces the need for extensive experimentation, potentially lowering development costs by up to 90%. The findings support WAAM’s applicability for complex components, such as multi-material or hybrid WAAM-subtractive builds, particularly for low-to-medium volume production in industries like aerospace. WAAM’s fatigue performance approaches 80–90% of traditional manufacturing (TM) counterparts (e.g., forged CT3 steel, N ≈ 385,042 cycles at equivalent σ), with the 10–20% gap due to layered microstructures and defects like porosity; our recommendations include post-treatments (e.g., machining to Ra < 0.05 mm, heat treatment, peening) and safety factors (~1.2) to bridge the TM gap.

The limitations of this study include the absence of quantitative defect analysis (e.g., porosity volume fraction via CT scans or advanced fractography), which could further link microstructural evolution (grain orientation, residual stresses, HAZs) to fatigue mechanisms. Future work should incorporate larger datasets for cross-validation, nonlinear models or response surface methodology (RSM) to capture all interactions, and full microstructural characterization (SEM, EBSD) to validate the 10–20% TM gap. Confirmation experiments at the optimal settings are recommended, alongside studies exploring additional factors like material properties, cooling rates, or advanced defect analysis.

## Figures and Tables

**Table 1:** Comparison of prior WAAM fatigue studies with the current work.

Study (Year)	Geometry	Material	Loading Mode	Parameters/Key Focus	Comparison to This Study
Williams et al. (2016) [2]	Planar orientations	Ti-6Al-4V	Fracture toughness, crack growth	Build direction, residual stress	Focuses on Ti crack propagation; this study focuses on steel cylindrical bending with Taguchi.
Li et al. (2022) [33]	Likely planar	308L SS	Low-cycle fatigue	Strain amplitudes, WAAM vs. cast	Comparative WAAM-cast; this study adds external cylindrical, high-cycle bending, and interactions.
Leuders et al. (2013) [45]	Not specified	TiAl6V4	Fatigue resistance	Porosity in SLM	Classic defects; this study extends to WAAM steel and the quantitative TM gap.
Brandl et al. (2011) [46]	Not specified	Ti-6Al-4V	Crack propagation	Defects simulation laser AM	Simulates porosity; this study simulates empirical bending and suggests CT future.
Günther et al. (2017) [34]	Not specified	Ti-6Al-4V	VHCF	SLM vs. EBM microstructure	VHCF regime; this study considers high-cycle bending and external WAAM.
Shunmugesh et al. (2025) [21]	Cylindrical	Low-carbon steel	Fabrication (not fatigue)	Travel speed, microstructure	Closest cylindrical; this study adds fatigue bending and parameter optimization.
Huang et al. (2023) [47,48]	Plates	Steel	Fatigue testing	Stress ratios, S-N diagrams	Planar focus; this study focuses on cylindrical external and process parameters.

**Table 2:** Process parameters and levels for WAAM fabrication.

Parameter	Level 1	Level 2	Level 3	Level 4	Level 5
Welding Current (I, A)	110	115	120	125	130
Offset Distance (O, mm)	1.5	1.875	2.25	2.625	3
Step Length (a, mm)	0 (Rotary)	25 (Spiral)	50 (Spiral)	75 (Spiral)	1000 (Straight)
Welding Speed (V, mm/min)	400	450	500	550	600
Gauge Diameter (d, mm)	16	16.25	16.5	16.75	17

**Table 3:** Nominal bending stress (σ) for each specimen gauge diameter (d).

**d (mm)**	16	16.25	16.5	16.75	17
**σ (MPa)**	54	52	49	47	45

**Table 4:** Average fatigue life (N) from the Taguchi L25 experiment.

Run Number	Weld Current I (A)	Offset Distance (mm)	Step Length a (mm)	Welding Speed V (mm/min)	Gauge Diameter d (mm)	Replicate 1	Replicate 2	Replicate 3	Mean of Fatigue Cycles (N)
1	110	2	0	400	16	209,607	235,364	225,586	223,519
2	110	2	25	450	16	262,210	240,291	240,290	247,597
3	110	2	50	500	17	245,479	289,080	274,778	269,779
4	110	3	75	550	17	297,797	257,981	312,962	289,580
5	110	3	1000	600	17	331,206	293,337	291,474	305,339
6	115	2	25	500	17	289,176	296,387	309,546	298,370
7	115	2	50	550	17	320,608	311,562	332,174	321,448
8	115	2	75	600	16	241,909	249,486	253,170	248,188
9	115	3	1000	400	16	269,677	287,514	255,781	270,991
10	115	3	0	450	17	294,491	298,979	267,637	287,036
11	120	2	25	600	16	273,145	250,735	245,326	256,402
12	120	2	50	400	16	269,745	270,641	262,228	267,538
13	120	2	75	450	17	288,631	276,546	310,800	291,992
14	120	3	1000	500	17	310,079	290,691	313,432	304,734
15	120	3	0	550	17	297,270	353,402	311,666	320,779
16	125	2	75	450	17	336,447	313,592	327,166	325,735
17	125	2	1000	500	16	258,652	239,856	280,616	259,708
18	125	2	0	550	16	264,152	273,003	270,598	269,251
19	125	3	25	600	17	296,308	315,273	266,489	292,690
20	125	3	50	400	17	313,727	304,281	321,832	313,280
21	130	2	1000	550	17	287,726	280,826	313,607	294,053
22	130	2	0	600	17	299,825	295,244	311,057	302,042
23	130	2	25	400	17	312,917	356,011	308,592	325,840
24	130	3	50	450	16	273,719	262,705	233,275	256,566
25	130	3	75	500	16	248,180	292,886	286,892	275,986

**Table 5:** Response table for delta values and ranking of factors.

Factor	Delta (dB)	Rank
d	4.00	1
I	0.80	2
O	0.69	3
V	0.62	4
a	0.24	5

**Table 6:** ANOVA table for the raw data.

Source	Sum of Squares	df	F-Value	*p*-Value	eta^2^	95% CI eta^2^
Welding current	1.98 × 10^10^	4	6.13	<0.001	0.13	[0.08–0.18]
Offset distance	8.45 × 10^9^	4	2.62	0.045	0.07	[0.03–0.11]
Step length	5.23 × 10^9^	4	1.62	0.182	0.04	[0.01–0.07]
Welding speed	4.12 × 10^9^	4	1.28	0.289	0.03	[0.00–0.06]
Gauge diameter	5.52 × 10^10^	4	171.62	<0.001	0.62	[0.55–0.68]
Residual	1.74 × 10^10^	54	-	-	-	-

**Table 7:** Mean S/N ratios for the interaction between welding current (I) and specimen gauge diameter (d).

Current/Gauge Diameter	16.00	16.25	16.50	16.75	17.00
110	106.99	107.87	108.62	109.24	109.70
115	107.90	108.66	109.16	109.50	109.50
120	108.18	108.55	109.31	109.68	110.12
125	108.29	108.60	109.33	109.92	110.26
130	108.18	108.82	109.37	109.60	110.26

**Table 8:** Mean S/N ratios for the interaction between specimen gauge diameter (d) and welding speed (V).

Gauge Diameter/Welding Speed	400	450	500	550	600
16.00	106.99	108.18	108.29		108.18
16.25	108.60	107.87	108.82	108.60	
16.50		109.23	108.62	109.37	109.33
16.75	109.59		109.59	109.24	109.60
17.00	110.26	110.26		110.13	109.70

**Table 9:** Mean S/N ratios for the interaction between welding current (I) and offset distance.

Welding Current/Offset Distance	1.500	1.875	2.250	2.625	3.000
110	106.99	107.87	108.62	109.24	109.70
115	109.50	109.50	107.90	108.66	109.16
120	108.18	108.55	109.31	109.68	110.12
125	110.26	108.29	108.60	109.33	109.92
130	109.37	109.60	110.26	108.18	108.82

**Table 10:** Fatigue life comparison for traditionally manufactured CT3 steel specimens.

Gauge Diameter (d, mm)	Fatigue Cycles (N) WAAM	Fatigue Cycles (N) Traditional
16	259,708	270,234
16.25	269,251	328,224
16.5	292,690	354,339
16.75	313,280	372,169
17	325,840	385,042

## Data Availability

The original contributions presented in this study are included in the article. Further inquiries can be directed to the corresponding author.

## Abbreviations

The following abbreviations are used in this manuscript:

AM	Additive Manufacturing
ANOVA	Analysis of Variance
ASTM	American Society for Testing and Materials
CI	Confidence Interval
CMT	Cold Metal Transfer
CNC	Computer Numerical Control
CT	Computed Tomography
CT3	Carbon Steel Type 3
CV	Coefficient of Variation
DC	Direct Current
EBM	Electron Beam Melting
EBSD	Electron Backscatter Diffraction
EDM	Electrical Discharge Machining
GTAW	Gas Tungsten Arc Welding
HAZ	Heat-Affected Zone
ISO	International Organization for Standardization
MIG	Metal Inert Gas
N	Mean of Fatigue Cycles
Ra	Arithmetic Average Roughness
RSM	Response Surface Methodology
Rz	Average Maximum Height of the Profile
S/N	Signal-to-Noise Ratio
SE	Standard Error
SEM	Scanning Electron Microscopy
SLM	Selective Laser Melting
TM	Traditional Manufacturing
VIF	Variance Inflation Factor
VHCF	Very High Cycle Fatigue
WAAM	Wire Arc Additive Manufacturing
WIM	Weigh-In-Motion
